# Supplementary material for: A Prospective, Real-World, Multinational Study of Naloxegol for Patients with Cancer Pain Diagnosed with Opioid-Induced Constipation—The NACASY Study
Source: Cancers (Basel). 2022 Feb 23;14(5):1128. doi: 10.3390/cancers14051128 (PMC8909554; doi:10.3390/cancers14051128)
Supplement: Supplementary file 1 [file cancers-14-01128-s001.zip › cancers-1598550-supplementary.pdf]

A Prospective, Real-World, Multinational Study of Naloxegol for Patients with Cancer Pain Diagnosed with Opioid-Induced Constipation—The NACASY Study

Andrew Davies, Saverio Cinieri, Denis Dupoiron, Sofia España Fernandez, Johan Leclerc, Vincenzo Montesarchio, Kyriaki Mystakidou, Judith Serna, Jan Tack and on behalf of the NACASY Study Group

Table S1. Study discontinuations.

**Reason for study discontinuation**	**N**	**%**
Naloxegol adverse reaction	12	21.4
Patient decision	10	17.9
Adverse event	8	14.3
Death	7	12.5
Loss to follow-up	7	12.5
Investigator decision	6	10.7
Consent withdrawn	6	10.7
**TOTAL**	**56**	**100**

Table S2: Opioid treatment modifications during the study.

		**Week 2**<br>**N (%)**	**Week 4**<br>**N (%)**
Treatment modified	Yes	31 (22.1)	22 (18.6)
	Total	140 (100)	118 (100)
Opioid treatment change	Dose increase	20 (64.5)	14 (63.6)
	Change of opioid	8 (25.8)	4 (18.2)
	Dose reduction	3 (9.7)	4 (18.2)
	Total	31 (100)	22 (100)

Table S3. Most common (≥5%) adverse events.

	Grade 1–3		Grade 4–5		Total	
	N	%	N	%	N	%
**Abdominal pain**	17	10.0	0	0.0	17	10.0
**Nausea**	15	8.8	0	0.0	15	8.8
**Anemia**	12	7.1	0	0.0	12	7.1
**Fatigue**	12	7.1	0	0.0	12	7.1
**Asthenia**	10	5.9	0	0.0	10	5.9
**Diarrhea**	9	5.3	0	0.0	9	5.3
**Vomiting**	9	5.3	0	0.0	9	5.3

Table S4. Changes in stool consistency according to the BSS

		Visit 1		Visit 2		Visit 3	
		N	%	N*	%	N**	%
**BSS-Stool consistency**	**1**	48	33.6	25	18.5	22	18.8
	**2**	24	16.8	24	17.8	25	21.4
	**3**	19	13.3	22	16.3	16	13.7
	**4**	13	9.1	26	19.3	23	19.7
	**5**	11	7.7	16	11.9	8	6.8
	**6**	3	2.1	12	8.9	8	6.8
	**7**	9	6.3	5	3.7	6	5.1
	**Not available**	16	11.2	5	3.7	9	7.7
	**Total**	143	100.0	135	100.0	117	100.0

*There were 8 patients with missing data

**There were 2 missing data

Note: There were 24 patients who have not completed visit 3

BSS: Bristol Stool Scale
